# Mapping the adoption and implementation of artificial intelligence (AI)-based conversational interface for tobacco cessation: a scoping review protocol

**DOI:** 10.1186/s13643-026-03140-x

**Published:** 2026-07-31

**Authors:** Navpreet Kaur, Priyobrat Rajkhowa, Shalini Bassi, Raj Mohan Panda, Monirujjaman Biswas, Mohai Menul Biswas, Monika Arora

**Affiliations:** 1 Public Health Foundation of India (PHFI), New Delhi, 110030 India; 2 HRIDAY, Green Park Extension, New Delhi, 110016 India

**Keywords:** Artificial intelligence, Consolidated Framework for Implementation Research (CFIR), Digital health interventions, Tobacco cessation

## Abstract

**Background:**

Tobacco use remains a leading global public health issue, accounting for nearly 8 million deaths annually. Despite ongoing efforts through various health interventions and programmes aimed at tobacco cessation, significant challenges persist, particularly in low- and middle-income countries (LMICs). The advent of artificial intelligence (AI)-driven conversational interfaces presents a promising, scalable approach to overcoming existing obstacles in traditional tobacco cessation initiatives. The aim of the scoping review is to systematically map and analyse the global evidence on the adoption and implementation of AI-based conversational interfaces for tobacco cessation. It will ultimately support strengthening the existing health system.

**Methods:**

This scoping review will follow the methodological framework outlined by Arksey and O’Malley and Levac et al. A comprehensive search will be conducted across four electronic databases: PubMed, CINAHL, Web of Science, and Scopus, using search terms related to AI-based conversational tools for tobacco cessation and implementation. The findings will be narratively synthesised, adopting the Consolidated Framework for Implementation Research (CFIR), structured according to its key domains. Review will follow the “Preferred Reporting Items for Systematic reviews and Meta-Analyses extension for Scoping Review (PRISMA-ScR)” guidelines.

**Conclusions/discussion:**

The review aims to identify the key barriers and facilitators for implementing AI-driven conversational interventions for tobacco cessation. It also seeks to identify best practices to support the effective integration of AI-driven cessation interventions across diverse healthcare contexts/settings. By mapping the global landscape and current evidence, particularly in the low-resource countries with high tobacco burden, this review will highlight the implementation challenges. Ultimately, this effort aims to support evidence-based deployment of AI-based conversational agents to achieve the WHO’s target to reduce the prevalence of tobacco use by < 5%.

**Systematic review registration:**

The protocol has been prospectively registered on the “Open Science Framework (OSF)”, https://doi.org/10.17605/OSF.IO/62FDB.

**Supplementary Information:**

The online version contains supplementary material available at https://doi.org/10.1186/s13643-026-03140-x.

## Introduction

Tobacco use presents a significant global public health challenge worldwide, contributing to millions of deaths each year and placing a substantial burden on the healthcare systems, as highlighted by multiple studies and the World Health Organization (WHO) [1, 2]. A striking 80% of the world’s 1.3 billion tobacco users reside in LMICs [1]. The prevalence of tobacco dependence is rising due to its chronic, relapsing–remitting characteristics [3].

The current traditional support to tobacco cessation, including cognitive behavioural therapy, pharmacotherapies, and nicotine replacement therapies (NRT), demonstrates varying levels of effectiveness [3–5]. However, these methods often encounter major limitations in helping quit tobacco use, such as inadequate accessibility, limited availability, poor adherence, and a lack of culturally relevant support, for both smoking and smokeless tobacco users [3, 5]. Reports indicate that over 60% of tobacco users are not offered adequate resources to assist in quitting, primarily due to constrained support systems within healthcare frameworks [6].

Digital platforms have enhanced access to tobacco cessation support, advancing from computer-generated letters to text messages and smartphone applications. However, they often lack the personalised help typically offered through therapist-led or group counselling sessions. Despite their effectiveness, such personalised interventions also remain underutilised, as highlighted in recent reviews [7, 8]. To address such gaps, emerging technologies, such as conversational AI, are being used. These conversational agent (CA) systems have demonstrated the ability to provide personalised care for tobacco cessation and other health conditions [7, 8]. Such CA interventions have been widely adopted across various sectors recently.

Conversational AI is described as “the study of techniques for creating software agents that can engage in natural conversational interactions with humans” [9]. This results in AI-empowered CAs, which are software systems that mimic interactions with humans [9].

The most common and emerging digital interventions are CAs which include chatbots, a text- and voice-based applications. Chatbots span from rule-based systems which rely on preprogrammed scripts to advanced LLMs utilising deep neural networks trained on large datasets enabling versatile, human-like conversational capabilities [10]. AI-based CAs are a growing area of research for smoking cessation that provide a human-like, personalised, 24/7, tailored approach with real-time support from advanced LLMs [2]. The current evidence suggests that the use of conversational AI-based agents for tobacco cessation has shown promising results and is an acceptable tool among the participants [3]. Aligning with the MPOWER framework “Offer help to quit”, this scoping review offers an opportunity to bridge the implementation gap in tobacco cessation, particularly in areas with limited counselling resources and healthcare workers’ reach [11].

However, with all the benefits of CAs using LLMs and promising potential in tobacco cessation, global adoption and implementation of AI-based CAs remain limited due to various factors. Since LLMs are trained on uncurated data sourced from the Internet, LLMs inherit and also amplify existing biases, misrepresentation, offensive language, and stereotypes. This causes social bias, affecting and harming the marginalised and vulnerable population [12]. Apart from privacy concerns, potential risk of misinformation, and heterogeneity in user needs, LLMs have been reported issues such as toxicity towards certain social groups, negative sentiments, stereotypical linguistic association, and poor recognition of certain language dialects [2, 7, 12]. To overcome these challenges and maximise the public health impact, it is crucial to understand the current landscape, identify barriers across diverse populations, and develop and implement scalable, accessible digital health solutions to meet specific user needs [2].

Therefore, the present scoping review aims to systematically map the current literature on the factors impacting the global adoption and implementation of AI-driven conversational interfaces for tobacco cessation. The findings will provide valuable insights to guide future research, policy development, and the development of scalable, evidence-based AI-based tobacco cessation interventions.

## Methodology

The current scoping review protocol is developed following the methodological framework outlined by Arksey and O’Malley and further refined by Levac et al. [13]. The review will follow the Preferred Reporting Items for Scoping Reviews (PRISMA-ScR) guidelines. This will adopt the “PRISMA-Pand Meta-Analysis Protocols 2015 checklist” to describe minimal recommended objects to address this scoping review protocol, which is given in Annexure 1 [14, 15]. The review will be conducted in the following stages:

### Stage 1: Identifying the review question

The team (M. A., S. B., R. P., P. R., N. K., M. B., M. M. B.) went through a process of brainstorming and refining ideas. An initial preliminary literature search on a global level was conducted to investigate the implementation of AI-based conversational interfaces for tobacco cessation. This led to the formulation and subsequent refinement of the primary research question by the team.

1. What factors influence the successful adoption and implementation of AI-based conversational interfaces for tobacco cessation among tobacco users and healthcare providers globally?

This study adopted the population, concept, and context (PCC) format, according to the JBI Manual for Evidence Synthesis 2020, for formulating the research question (Table 1) [14].

**Table 1:** PCC framework for developing the research question

Sl. no.	Concept	Details
1	Population (P)	Tobacco users — smoking and smokeless tobacco users
2	Concept (C)	Implementation of AI-based conversational interfaces for tobacco cessation
3	Context (C)	Global

### Stage 2: Identifying relevant studies

To identify relevant studies, the research question will be divided into its core concepts, and keywords will be identified for each concept. The eligible studies will be identified using a combination of keywords such as “smoker”, “smokeless tobacco user*”, “nicotine user*”, “chewing tobacco”, “tobacco cessation”, “smoking cessation”, “behavioural therapy”, “motivational intervention”, “machine learning”, “conversational agents”, and “chatbots” on a global scale. Boolean operators such as AND/OR will be used to merge keywords effectively. The search will be restricted to articles published in English. A comprehensive search will be conducted across multiple databases, including PubMed, CINAHL, Web of Science, and Scopus, to identify relevant literature. A reference overview of the electronic search from one selected database (PubMed) is included in Annexure 2. Furthermore, additional eligible articles will be identified by reviewing the reference lists of included studies. Data management will be carried out using Rayyan, a systematic review web-based management platform [16].

### Stage 3: Study selection

All identified citations will be collected and uploaded into Rayyan, with duplicates removed after the search. Three reviewers (N. K., M. B., and M. M. B.) will independently screen all retrieved titles and abstracts against the predefined inclusion and exclusion criteria (Table 2). Any conflicts regarding eligibility will be resolved by a fourth reviewer (P. R.), who will make the final decision. All authors will collectively agree on potentially relevant research, and full texts will be retrieved accordingly.

**Table 2:** Inclusion and Exclusion Criteria

Sl. no	Inclusion	Exclusion
1	Studies focus on adoption and implementation components such as feasibility, acceptability, user engagement, and usability of AI-based conversational interfaces for tobacco cessation	Studies focus on the implementation of human-led behavioural pharmacotherapy for tobacco cessation. Studies focus on the development process of AI-based conversational interfaces
2	Studies published in English	Non-English language studies will be excluded
3	Original studies such as interventional studies, cross-sectional studies (quantitative, mixed method), qualitative studies, and secondary data analysis	Reviews, study protocol, conference abstracts, newspaper blogs
4	Time period: Studies from January 2015 to April 2025	Studies published before 2015 will be excluded

The full texts of studies that pass the initial title and abstract screening will be independently reviewed by the same three reviewers (N. K., M. B., and M. M. B.). If the full texts of the sources of evidence do not meet the inclusion criteria, the reviewers will document and explain the reasons for exclusion. The studies that meet all inclusion criteria will be considered for final analysis. The final scoping review will feature a comprehensive report on the search outcomes and the methodology for included studies, which will be illustrated by adopting the PRISMA 2020 flow diagram [17].

### Stage 4: Charting the data

Data charting will be systematically conducted using a predefined data charting form (Annexure 3). Each eligible paper will undergo independent data charting by three reviewers (N. K., M. B., and M. M. B.) using the data charting form. The data charting will comprehensively include details about the participants, context, concept, study methods, facilitators, barriers, and critically relevant findings to the review questions. To achieve the objective, the data extraction form will specifically collect the following details from each included study, namely (i) title, (ii) author, (iii) publication year, (iv) study setting, (v) aims and objectives, (vi) country where the research was conducted, (vii) study design, (viii) participants, (ix) results, (x) findings pertinent to the review question, (xi) conclusion, and (xii) recommendations. Along with the retrieved data, narrative data will be gathered to provide a richer understanding of the research question. This will include information on the following: Countries where AI-based conversational agents for tobacco cessation have been adopted and implemented, specific methods employed in their adoption and implementation, and detailed reasons and factors that hinder or facilitate their adoption and implementation. The authors of the papers will be contacted if necessary to obtain any additional or missing information. This comprehensive data charting procedure is designed to facilitate the identification of research gaps within the field.

### Stage 5: Collating, summarising, and reporting the results

The study findings will be summarised and reported through a thematic synthesis that adopts the CFIR framework. The CFIR has been widely employed in numerous studies, especially within the realm of mobile health (mHealth) solutions, to assess factors influencing the adoption and implementation of mHealth applications. Its utilisation offers a robust foundation for this scoping review and facilitates a systematic analysis of study outcomes [18]. The CFIR framework comprises five major domains. The first domain, intervention characteristics, concentrates on the properties of the intervention itself. The second domain, the outer setting, includes factors in the broader external environment that may influence the implementation process. The third domain, inner setting, comprises elements within the organisation or setting where the intervention will be implemented. The fourth domain, individuals, considers the characteristics of the individuals involved in the implementation process. Finally, the fifth domain, the implementation process, focuses on the specific strategies and methods used to implement the intervention. In addition to the narrative analysis, various tables will illustrate the characteristics and findings of the included studies [19].

#### Expected results

The study is expected to systematically identify and synthesise existing evidence on the factors influencing the implementation and adoption of the AI-based conversational interface for quitting both smoking and smokeless tobacco on a global level.

#### Registration

The protocol has been prospectively registered on the “Open Science Framework (OSF)”, DOI: https://doi.org/10.17605/OSF.IO/62FDB.

## Discussion

This scoping review aims to systematically map the global evidence and experiences related to the adoption and implementation of AI-based conversational agents for tobacco cessation worldwide. In addition, the review will explore the various factors that influence the incorporation of these technologies into healthcare systems. The study will examine factors such as design acceptance, user digital literacy, adaptability, infrastructure readiness, and real-world challenges in LMIC and higher-middle-income countries and settings. These components and factors will be assessed through CFIR’s domains, including intervention characteristics, inner and outer settings, characteristics of individuals, and the implementation process.

The increasing interest in AI for digital health interventions offers both opportunities and challenges. This review aims to identify gaps, successes, and context-specific enablers or barriers that influence the scale-up and sustainability of conversational agents in tobacco cessation efforts for both smoking and smokeless tobacco. By analysing global implementation experiences and contextual considerations, the findings aim to offer empirical insights that can inform future design, deployment, and policy formulation related to digital cessation tools.

In addition, this review will assist healthcare stakeholders, policymakers, and developers aiming to integrate AI-based interventions into current public health strategies. The findings can inform scalable approaches to digital tobacco cessation, especially in settings with limited access and resources to implement conventional cessation services. Finally, the review aims to contribute to building more responsive, technology-enabled public health systems by capturing practical lessons and informing best practices for effective and sustainable AI integration in behavioural health interventions.

## Conclusion

The findings of this review will be instrumental in shaping supportive environments that promote the ethical and sustainable integration of AI into public health strategies for cessation. This encompasses critical considerations such as digital infrastructure, data privacy, workforce readiness, and equitable access. Ultimately, the review is expected to support evidence-informed decision-making for governments, health systems, and implementation partners striving to leverage AI technologies to reduce tobacco use and associated health burdens.

## Supplementary Information

Supplementary Material 1. Annexures 1,2, and 3

## Data Availability

Data sharing is not applicable to this study.

## Abbreviations

AI: Artificial intelligence
CA: Conversational agents
CFIR: Consolidated Framework for Implementation Research
LMICs: Low- and middle-income countries
NRT: Nicotine replacement therapies
PCC: Population, concept, and context
PRISMA-ScR: Preferred Reporting Items for Systematic reviews and Meta-Analyses extension for Scoping Reviews
WHO: World Health Organization

## References

[1] World Health Organisation. Tobacco. Available from: https://www.who.int/news-room/fact-sheets/detail/tobacco. Accessed 26 May 2025.

[2] Alkhudhairy RA, Gaikwad RN, Aljubua RH, Alharbi AM. The role of chatbots in tobacco cessation: a scoping review of applications, benefits, and challenges. J Neonatal Surg 2025;14 (7):950–9. Accessed 13 Oct 2025

[3] Bendotti H, Lawler S, Ireland D, Gartner C, Marshall HM. Co-designing a smoking cessation chatbot: focus group study of end users and smoking cessation professionals. JMIR Hum Factors. 2024;11:e56505. 10.2196/56505. Accessed 13 Oct 2025. PMC11369547 39159451

[4] Deepika V, Jodalli PS, Avinash BR. The role of chatbots and virtual assistants in enhancing tobacco cessation counselling. Front Digit Health. 2025;7:1503227. 10.3389/fdgth.2025. Accessed 13 Oct 2025. PMC12040952 40309319

[5] Nadkarni A, Gaikwad L, Sequeira M, D’souza J, Lopes M, Haldankar R, et al. Text message-based cessation intervention for people who smoked or used smokeless tobacco in India: a feasibility randomized controlled trial. Nicotine Tob Res. 2024;26(9):1201–8. 10.1093/ntr/ntae056. Accessed 13 Oct 2025. PMC11339165 38468498

[6] WHO releases first-ever clinical treatment guideline for tobacco cessation in adults. Available from: https://www.who.int/news/item/02-07-2024-who-releases-first-ever-clinical-treatment-guideline-for-tobacco-cessation-in-adults. Accessed 13 Oct 2025. PMC11288497 39074891

[7] Bendotti H, Lawler S, Chan G, Gartner C, Ireland D, Marshall H. Conversational artificial intelligence interventions to support smoking cessation: A systematic review and meta-analysis. Digit Health 2023;9. 10.1177/20552076231211634. Accessed 29 Jan 2026. PMC10623979 37928336

[8] He L, Balaji D, Wiers RW, Antheunis ML, Krahmer E. Effectiveness and acceptability of conversational agents for smoking cessation: a systematic review and meta-analysis. Nicotine Tob Res. 2023;25(7):1241–50. 10.1093/ntr/ntac281. Accessed 29 Jan 2026. PMC10256885 36507916

[9] Mariani MM, Hashemi N, Wirtz J. Artificial intelligence empowered conversational agents: a systematic literature review and research agenda. J Bus Res. 2023;161:113838. 10.1016/j.jbusres.2023.113838. Accessed 29 Jan 2026.

[10] Hua Y, Siddals S, Ma Z, Galatzer‐Levy I, Xia W, Hau C, et al. Charting the evolution of artificial intelligence mental health chatbots from rule‐based systems to large language models: a systematic review. World Psychiatry. 2025;24:383–94. 10.1002/wps.21352. Accessed 29 Jan 2026. PMC12434366 40948070

[11] Tata Institute of Social Sciences (TISS), Mumbai and Ministry of Health and Family Welfare, Government of India. GLOBAL ADULT TOBACCO SURVEY,INDIA 2016–2017 | Report. Available from: https://ntcp.mohfw.gov.in/assets/document/surveys-reports-publications/Global-Adult-Tobacco-Survey-Second-Round-India-2016-2017.pdf. Accessed 13 Oct 2025.

[12] Gallegos IO, Rossi RA, Barrow J, Tanjim MM, Kim S, Dernoncourt F, et al. Bias and fairness in large language models: a survey. Comput Linguist. 2026;2024(50):1097–179. 10.1162/coli_a_00524.AccessedJanuary29. Accessed 29 Jan 2026.

[13] Arksey H, O’Malley L. Scoping studies: towards a methodological framework. Int J Soc Res Methodol. 2005;8(1):19–32. 10.1080/1364557032000119616. Accessed 13 Oct 2025.

[14] Peters MDJ, Marnie C, Tricco AC, Pollock D, Munn Z, Alexander L, et al. Updated methodological guidance for the conduct of scoping reviews. JBI Evid Synth. 2020;18:2119. 10.11124/JBIES-20-00167. Accessed 13 Oct 2025. 33038124

[15] Shamseer L, Moher D, Clarke M, Ghersi D, Liberati A, Petticrew M, et al. Preferred Reporting Items for Systematic review and Meta-Analysis Protocols (PRISMA-P) 2015: elaboration and explanation. BMJ. 2015;349:g7647. 10.1136/bmj.g7647. Accessed 13 Oct 2025. 25555855

[16] Ouzzani M, Hammady H, Fedorowicz Z, Elmagarmid A. Rayyan—a web and mobile app for systematic reviews. Syst Rev. 2016;5(1):210. 10.1186/s13643-016-0384-4. Accessed 13 Oct 2025. PMC5139140 27919275

[17] Page MJ, McKenzie JE, Bossuyt PM, Boutron I, Hoffmann TC, Mulrow CD, et al. The PRISMA 2020 statement: an updated guideline for reporting systematic reviews. BMJ 2021;372:n71. 10.1136/bmj.n71. Accessed 13 Oct 2025. PMC8005924 33782057

[18] Ardito V, Golubev G, Ciani O, Tarricone R. Evaluating barriers and facilitators to the uptake of mHealth apps in cancer care using the consolidated framework for implementation research: scoping literature review. JMIR Cancer. 2023;9:e42092. 10.2196/42092. Accessed 13 Oct 2025. PMC10131717 36995750

[19] Damschroder LJ, Aron DC, Keith RE, Kirsh SR, Alexander JA, Lowery JC. Fostering implementation of health services research findings into practice: a consolidated framework for advancing implementation science. Implement Sci. 2009;4(1):50. 10.1186/1748-5908-4-50. Accessed 13 Oct 2025. PMC2736161 19664226

