# Supplementary material for: Mapping the adoption and implementation of artificial intelligence (AI)-based conversational interface for tobacco cessation: a scoping review protocol
Source: Syst Rev. 2026 Jul 31;15:279. doi: 10.1186/s13643-026-03140-x (PMC13685209; doi:10.1186/s13643-026-03140-x)
Supplement: Supplementary file 1 [file 13643_2026_3140_MOESM1_ESM.docx]

**Annexures**

**Annexure 1**

**PRISMA-P (Preferred Reporting Items for Systematic review and Meta-Analysis Protocols) 2015 checklist: recommended items to address in a systematic review protocol***

| Section and topic | Item No | Checklist item | Page Number |
| --- | --- | --- | --- |
| ADMINISTRATIVE INFORMATION | | |  |
| Title: |  |  |  |
| Identification | 1a | Identify the report as a protocol of a systematic review | Page number: 1 |
| Update | 1b | If the protocol is for an update of a previous systematic review, identify as such | N/A |
| Registration | 2 | If registered, provide the name of the registry (such as PROSPERO) and registration number | Page number: 1  Open Science Framework (OSF)”, DOI: 10.17605/OSF.IO/62FDB |
| Authors: |  |  |  |
| Contact | 3a | Provide name, institutional affiliation, e-mail address of all protocol authors; provide physical mailing address of corresponding author | Page number: 1 |
| Contributions | 3b | Describe contributions of protocol authors and identify the guarantor of the review | Page number: 5 |
| Amendments | 4 | If the protocol represents an amendment of a previously completed or published protocol, identify as such and list changes; otherwise, state plan for documenting important protocol amendments | N/A |
| Support: |  |  |  |
| Sources | 5a | Indicate sources of financial or other support for the review | Page number: 5 |
| Sponsor | 5b | Provide name for the review funder and/or sponsor | Page number: 5 |
| Role of sponsor or funder | 5c | Describe roles of funder(s), sponsor(s), and/or institution(s), if any, in developing the protocol | Page number: 5 |
| INTRODUCTION | | |  |
| Rationale | 6 | Describe the rationale for the review in the context of what is already known | Page number: 2 |
| Objectives | 7 | Provide an explicit statement of the question(s) the review will address with reference to participants, interventions, comparators, and outcomes (PICO) | Page number: 3 |
| METHODS | | |  |
| Eligibility criteria | 8 | Specify the study characteristics (such as PICO, study design, setting, time frame) and report characteristics (such as years considered, language, publication status) to be used as criteria for eligibility for the review | Adopted PCC, Page number: 3 |
| Information sources | 9 | Describe all intended information sources (such as electronic databases, contact with study authors, trial registers or other grey literature sources) with planned dates of coverage | Page number: 3 |
| Search strategy | 10 | Present draft of search strategy to be used for at least one electronic database, including planned limits, such that it could be repeated | Annex 2, Page number: 4-6 |
| Study records: |  |  |  |
| Data management | 11a | Describe the mechanism(s) that will be used to manage records and data throughout the review | Page number: 3 |
| Selection process | 11b | State the process that will be used for selecting studies (such as two independent reviewers) through each phase of the review (that is, screening, eligibility and inclusion in meta-analysis) | Page number: 3,4 |
| Data collection process | 11c | Describe planned method of extracting data from reports (such as piloting forms, done independently, in duplicate), any processes for obtaining and confirming data from investigators | Annex-3, Page number:7-8 |
| Data items | 12 | List and define all variables for which data will be sought (such as PCC items, funding sources), any pre-planned data assumptions and simplifications | Page number: 3 |
| Outcomes and prioritization | 13 | List and define all outcomes for which data will be sought, including prioritization of main and additional outcomes, with rationale | Annex 3, Page number: 7-8 |
| Risk of bias in individual studies | 14 | Describe anticipated methods for assessing risk of bias of individual studies, including whether this will be done at the outcome or study level, or both; state how this information will be used in data synthesis | N/A |
| Data synthesis | 15a | Describe criteria under which study data will be quantitatively synthesised | Thematic synthesis |
|  | 15b | If data are appropriate for quantitative synthesis, describe planned summary measures, methods of handling data and methods of combining data from studies, including any planned exploration of consistency (such as I^2^, Kendall’s τ) | N/A |
|  | 15c | Describe any proposed additional analyses (such as sensitivity or subgroup analyses, meta-regression) | N/A |
|  | 15d | If quantitative synthesis is not appropriate, describe the type of summary planned | N/A |
| Meta-bias(es) | 16 | Specify any planned assessment of meta-bias(es) (such as publication bias across studies, selective reporting within studies) | N/A |
| Confidence in cumulative evidence | 17 | Describe how the strength of the body of evidence will be assessed (such as GRADE) | N/A |

*** It is strongly recommended that this checklist be read in conjunction with the PRISMA-P Explanation and Elaboration (cite when available) for important clarification on the items. Amendments to a review protocol should be tracked and dated. The copyright for PRISMA-P (including checklist) is held by the PRISMA-P Group and is distributed under a Creative Commons Attribution Licence 4.0.**

*From: Shamseer L, Moher D, Clarke M, Ghersi D, Liberati A, Petticrew M, Shekelle P, Stewart L, PRISMA-P Group. Preferred reporting items for systematic review and meta-analysis protocols (PRISMA-P) 2015: elaboration and explanation. BMJ. 2015 Jan 2;349(jan02 1):g7647.*

**Annexure 2**

| **Annexure 2** | | | |
| --- | --- | --- | --- |
| **Search Strategy: PubMed, Date 11/09/2025** | | | |
| **Sl. No** | **Concept** | **Search Strategy** | **Hits** |
| a. | Cessation with AI | ("cessation"[Title/Abstract] OR ("stop*"[Title/Abstract] OR "end"[Title/Abstract]) OR ("Tobacco Use Cessation"[MeSH Terms] OR ("Tobacco Use Cessation Devices"[MeSH Terms] OR "Tobacco Control"[MeSH Terms]))) AND ("Artificial Intelligence"[Title/Abstract] OR "AI"[Title/Abstract] OR "machine learning"[Title/Abstract] OR "deep learning"[Title/Abstract] OR "neural network*"[Title/Abstract] OR "natural language processing"[Title/Abstract] OR "NLP"[Title/Abstract] OR "automated system*"[Title/Abstract] OR "intelligent system*"[Title/Abstract] OR "computational intelligence"[Title/Abstract] OR "predictive modeling"[Title/Abstract] OR "ai powered system*"[Title/Abstract] OR "ai driven intervention*"[Title/Abstract] OR "cognitive computing"[Title/Abstract] OR "data driven model*"[Title/Abstract] OR "automated decision-making"[Title/Abstract] OR "artificial neural network*"[Title/Abstract] OR "ai based algorithm*"[Title/Abstract] OR "AI-assisted"[Title/Abstract] OR "intelligent automation"[Title/Abstract] OR "self learning algorithm*"[Title/Abstract] OR "reinforcement learning"[Title/Abstract] OR "chatbot*"[Title/Abstract] OR "virtual assistant*"[Title/Abstract] OR "conversational agent*"[Title/Abstract] OR "Generative Artificial Intelligence"[MeSH Terms] OR "Artificial Intelligence"[MeSH Terms]) | 23,215 |
| b. | Tobacco User/Use | "tobacco user"[Title/Abstract] OR "smoker*"[Title/Abstract] OR "smokeless tobacco user*"[Title/Abstract] OR "nicotine user*"[Title/Abstract] OR "nicotine addict*"[Title/Abstract] OR "tobacco addict*"[Title/Abstract] OR "tobacco"[Title/Abstract] OR "Tobacco dependent"[Title/Abstract] OR "Tobacco Smokeless"[Title/Abstract] OR "Smokeless Tobacco"[Title/Abstract] OR "Gutkha"[Title/Abstract] OR "Khaini"[Title/Abstract] OR "Zarda"[Title/Abstract] OR "Pan Masala"[Title/Abstract] OR "snuff"[Title/Abstract] OR "snus"[Title/Abstract] OR "dipping tobacco"[Title/Abstract] OR "oral tobacco"[Title/Abstract] OR "SLT user"[All Fields] OR "tobacco cessation"[Title/Abstract] OR "smoking cessation"[Title/Abstract] OR "quit smoking"[Title/Abstract] OR "stop smoking"[Title/Abstract] OR "Tobacco Use Cessation"[Title/Abstract] OR "nicotine cessation"[Title/Abstract] OR "tobacco quitting"[Title/Abstract] OR "quitting smoking"[Title/Abstract] OR "giving up smoking"[Title/Abstract] OR "smoking reduction"[Title/Abstract] OR "tobacco dependence treatment"[Title/Abstract] OR "nicotine dependence treatment"[Title/Abstract] OR "smoking intervention"[Title/Abstract] OR "tobacco intervention"[Title/Abstract] OR "quit attempt*"[Title/Abstract] OR "smoking relapse prevention"[Title/Abstract] OR "tobacco, smokeless"[MeSH Terms] OR "Tobacco Products"[MeSH Terms] OR "Tobacco Use"[MeSH Terms] OR "Tobacco Use Disorder"[MeSH Terms] OR "Smokers"[MeSH Terms] | 238,959 |
| c. | Combined a and b (Tobacco cessation AND AI) | ("cessation"[Title/Abstract] OR ("stop*"[Title/Abstract] OR "end"[Title/Abstract]) OR ("Tobacco Use Cessation"[MeSH Terms] OR ("Tobacco Use Cessation Devices"[MeSH Terms] OR "Tobacco Control"[MeSH Terms]))) AND ("Artificial Intelligence"[Title/Abstract] OR "AI"[Title/Abstract] OR "machine learning"[Title/Abstract] OR "deep learning"[Title/Abstract] OR "neural network*"[Title/Abstract] OR "natural language processing"[Title/Abstract] OR "NLP"[Title/Abstract] OR "automated system*"[Title/Abstract] OR "intelligent system*"[Title/Abstract] OR "computational intelligence"[Title/Abstract] OR "predictive modeling"[Title/Abstract] OR "ai powered system*"[Title/Abstract] OR "ai driven intervention*"[Title/Abstract] OR "cognitive computing"[Title/Abstract] OR "data driven model*"[Title/Abstract] OR "automated decision-making"[Title/Abstract] OR "artificial neural network*"[Title/Abstract] OR "ai based algorithm*"[Title/Abstract] OR "AI-assisted"[Title/Abstract] OR "intelligent automation"[Title/Abstract] OR "self learning algorithm*"[Title/Abstract] OR "reinforcement learning"[Title/Abstract] OR "chatbot*"[Title/Abstract] OR "virtual assistant*"[Title/Abstract] OR "conversational agent*"[Title/Abstract] OR "Generative Artificial Intelligence"[MeSH Terms] OR "Artificial Intelligence"[MeSH Terms]) AND ("tobacco user"[Title/Abstract] OR "smoker*"[Title/Abstract] OR "smokeless tobacco user*"[Title/Abstract] OR "nicotine user*"[Title/Abstract] OR "nicotine addict*"[Title/Abstract] OR "tobacco addict*"[Title/Abstract] OR "tobacco"[Title/Abstract] OR "Tobacco dependent"[Title/Abstract] OR "Tobacco Smokeless"[Title/Abstract] OR "Smokeless Tobacco"[Title/Abstract] OR "Gutkha"[Title/Abstract] OR "Khaini"[Title/Abstract] OR "Zarda"[Title/Abstract] OR "Pan Masala"[Title/Abstract] OR "snuff"[Title/Abstract] OR "snus"[Title/Abstract] OR "dipping tobacco"[Title/Abstract] OR "oral tobacco"[Title/Abstract] OR "SLT user"[All Fields] OR "tobacco cessation"[Title/Abstract] OR "smoking cessation"[Title/Abstract] OR "quit smoking"[Title/Abstract] OR "stop smoking"[Title/Abstract] OR "Tobacco Use Cessation"[Title/Abstract] OR "nicotine cessation"[Title/Abstract] OR "tobacco quitting"[Title/Abstract] OR "quitting smoking"[Title/Abstract] OR "giving up smoking"[Title/Abstract] OR "smoking reduction"[Title/Abstract] OR "tobacco dependence treatment"[Title/Abstract] OR "nicotine dependence treatment"[Title/Abstract] OR "smoking intervention"[Title/Abstract] OR "tobacco intervention"[Title/Abstract] OR "quit attempt*"[Title/Abstract] OR "smoking relapse prevention"[Title/Abstract] OR ("tobacco, smokeless"[MeSH Terms] OR "Tobacco Products"[MeSH Terms] OR ("Tobacco Use"[MeSH Terms] OR "Tobacco Use Disorder"[MeSH Terms] OR "Smokers"[MeSH Terms]))) | 363 |

**Annexure 3**

| **Data charting form** | | |
| --- | --- | --- |
| **Section** | **Field** | **Description** |
| **Study Identification** | Study ID | Unique identifier (e.g., S001) |
|  | Title | Full title of the article |
|  | Author(s) | First author and et al. |
|  | Year of Publication | Year when the study was published |
|  | Country/Region | Where the study was conducted |
|  | World Bank Income Category | HIC / UMIC / LMIC / LIC |
|  | Type of Publication | Journal article, grey literature, report |
| **Study Characteristics** | Study Design | Qualitative, Quantitative, Mixed-methods, Pilot, RCT |
|  | Aim/Objectives | As stated by authors |
|  | Sample Size | Total number of participants |
|  | Participant Type | Tobacco users, healthcare providers, others |
|  | Participant Characteristics | Age, sex, education, digital literacy |
|  | Setting | Community, primary care, hospital, digital-only, etc. |
| **Intervention Characteristics** | Name of Intervention | e.g., QuitBot, SmokeFreeApp |
|  | Type of AI Tool | Chatbot, Virtual Assistant, LLM-based tool |
|  | AI Features Used | NLP, ML, voice interaction, personalization |
|  | Mode of Delivery | SMS, mobile app, web-based, wearable, etc. |
|  | Duration of Use | Weeks/months of usage |
|  | Frequency of Use | Number of interactions or sessions |
|  | Integration with Health System | Standalone or embedded in service delivery |
| **Implementation Factors (CFIR)** | **Intervention Characteristics**: Source of Evidence | Authoritative source or local innovation |
|  | Adaptability | Was the intervention adapted to the local context? (Y/N) |
|  | Complexity | High/Medium/Low based on user or provider burden |
|  | Cost and Resource Needs | High/Low/Not Reported |
|  | **Outer Setting**: Patient Needs & Resources | Customization, ease of access |
|  | External Incentives | National digital health policies, donor support |
|  | Socio-cultural Context | Relevance of content to local culture, language barriers |
|  | **Inner Setting**: Health System Readiness | Digital infrastructure, data systems, interoperability |
|  | Organizational Support | Leadership, IT support, provider training |
|  | Implementation Climate | Readiness for innovation, openness to AI tools |
|  | **Individual Characteristics**: User Digital Literacy | Low/Moderate/High |
|  | User Motivation to Quit | Captured or not captured |
|  | Healthcare Provider Perceptions | Facilitators/barriers from providers |
|  | Engagement and Retention | Completion rates, dropout reasons |
|  | **Implementation Process:** Planning Activities | Pilot testing, stakeholder mapping |
|  | Execution Strategy | Phased rollout, parallel arms |
|  | Support Mechanisms | Technical helpdesk, peer support, reminders |
|  | Evaluation Strategy | Indicators used, frequency of monitoring |
| **Outcomes and Impact** | Primary Outcomes | Tobacco cessation rates, reduction in use |
|  | Secondary Outcomes | Satisfaction, usability, engagement, app retention |
|  | Implementation Outcomes | Acceptability, feasibility, fidelity, scalability |
|  | Cost-Effectiveness | Mentioned or not, brief summary |
|  | Long-term Follow-up | Yes/No and duration |
| **Barriers and Facilitators** | Reported Barriers | E.g., digital illiteracy, lack of motivation, technical glitches |
|  | Reported Facilitators | E.g., personalization, ease of access, provider endorsement |
|  | Lessons Learned | As mentioned by authors |
|  | Recommendations | Policy or practice suggestions from the authors |
| **Notes and Additional Info** | Ethical Considerations | Informed consent, data privacy |
|  | Missing Information | Details not reported in the study |
|  | Contacted Authors | Yes/No and outcome of contact |
|  | Other Comments | Any relevant information not captured above |
|  | Other Key findings | (If any) |
|  | Conclusion | (If any) |
|  | Decision | (Include/Exclude/Maybe) |
|  | Reason | (In the case of exclusion) |
